# Cereal asparagine synthetase genes

**DOI:** 10.1111/aab.12632

**Published:** 2020-09-04

**Authors:** Sarah Raffan, Nigel G. Halford

**Affiliations:** ^1^ Plant Sciences Department Rothamsted Research Harpenden UK

**Keywords:** acrylamide, amino acids, asparagine metabolism, asparagine synthetase, barley, brachypodium, food safety, gene family evolution, maize, rice, rye, sorghum, wheat, wheat evolution

## Abstract

Asparagine synthetase catalyses the transfer of an amino group from glutamine to aspartate to form glutamate and asparagine. The accumulation of free (nonprotein) asparagine in crops has implications for food safety because free asparagine is the precursor for acrylamide, a carcinogenic contaminant that forms during high‐temperature cooking and processing. Here we review publicly available genome data for asparagine synthetase genes from species of the Pooideae subfamily, including bread wheat and related wheat species (*Triticum* and *Aegilops* spp.), barley (*Hordeum vulgare*) and rye (*Secale cereale*) of the Triticeae tribe. Also from the Pooideae subfamily: brachypodium (*Brachypodium dIstachyon*) of the Brachypodiae tribe. More diverse species are also included, comprising sorghum (*Sorghum bicolor*) and maize (*Zea mays*) of the Panicoideae subfamily and rice (*Oryza sativa*) of the Ehrhartoideae subfamily. The asparagine synthetase gene families of the Triticeae species each comprise five genes per genome, with the genes assigned to four groups: 1, 2, 3 (subdivided into 3.1 and 3.2) and 4. Each species has a single gene per genome in each group, except that some bread wheat varieties (genomes AABBDD) and emmer wheat (*Triticum dicoccoides*; genomes AABB) lack a group 2 gene in the B genome. This raises questions about the ancestry of cultivated pasta wheat and the B genome donor of bread wheat, suggesting that the hybridisation event that gave rise to hexaploid bread wheat occurred more than once. In phylogenetic analyses, genes from the other species cluster with the Triticeae genes, but brachypodium, sorghum and maize lack a group 2 gene, while rice has only two genes, one group 3 and one group 4. This means that *TaASN2*, the most highly expressed asparagine synthetase gene in wheat grain, has no equivalent in maize, rice, sorghum or brachypodium. An evolutionary pathway is proposed in which a series of gene duplications gave rise to the five genes found in modern Triticeae species.

## Graphical abstract

This article reviews the asparagine synthetase gene families of several wheat species, rye, barley, brachypodium, sorghum, maize and rice. It shows that *TaASN2*, the most highly expressed asparagine synthetase gene in wheat grain, has no equivalent in maize, rice, sorghum or brachypodium. It also raises questions about the ancestry of cultivated pasta and bread wheat. The review enabled an evolutionary pathway to be drawn up in which a series of gene duplications gave rise to the five genes found in modern Triticeae species. Asparagine synthetase is important because free asparagine is the precursor for acrylamide, a carcinogenic processing contaminant that forms during frying, baking, roasting and toasting of products made from cereal grains, as well the tubers, beans and storage roots of other crop species.

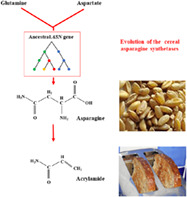

## 1 INTRODUCTION

Asparagine is an important nitrogen transport and storage molecule in many plant species (Lea, Sodek, Parry, Shewry, & Halford, 2007). In most plant tissues it is a major component of the free (soluble, nonprotein) amino acid pool and, together with glutamine and glutamate, it accounts for up to 70% of free amino acid content in wheat grain (Curtis et al., 2009). It reaches high concentrations during seed germination and in response to different biotic and abiotic stresses (Lea et al., 2007). It is also, of course, one of the amino acids used to make proteins.

Interest in the synthesis, accumulation and breakdown of asparagine in crop plants has been stimulated in recent years because of the discovery that free asparagine is the precursor for acrylamide formation. Acrylamide forms during the frying, baking, roasting, toasting and high‐temperature processing of grains, tubers, storage roots, beans and other crop products (Mottram, Wedzicha, & Dodson, 2002; Stadler et al., 2002; Zyzak et al., 2003; reviewed by Raffan & Halford, 2019). It is classed as a group 2a carcinogen by the International Agency for Research on Cancer (IARC, 1994) and in 2015 the European Food Safety Authority (EFSA) Expert Panel on Contaminants in the Food Chain (CONTAM) concluded that the margins of exposure to dietary acrylamide indicated “a concern for neoplastic effects” (CONTAM Panel, 2015). Subsequently (April 2018), Commission Regulation (EU) 2017/2158 came into force across the European Union, introducing compulsory risk management measures that apply to all food businesses (European Commission, 2017).

The development of crop varieties with reduced acrylamide‐forming potential will require greater knowledge and understanding of the genetic control of asparagine metabolism. In wheat grain, free asparagine accumulates to very high levels in response to sulphur deficiency (Curtis et al., 2009; Curtis, Powers, Wang, & Halford, 2018; Granvogl, Wieser, Koehler, Von Tucher, & Schieberle, 2007; Muttucumaru et al., 2006) and poor disease control (Curtis, Powers, & Halford, 2016; Martinek et al., 2009), while nitrogen fertilisation, in contrast to sulphur, also promotes free asparagine accumulation (Claus et al., 2006; Martinek et al., 2009). There are also substantial differences in the free asparagine concentration of grain from different wheat and rye varieties and genotypes (Curtis et al., 2010; Curtis, Powers, et al., 2018; Postles, Powers, Elmore, Mottram, & Halford, 2013). A complex network has been drawn up to describe the genes, enzymes, metabolites and environmental factors that affect asparagine metabolism (Curtis, Bo, Tucker, & Halford, 2018), but most attention to date has been focussed on the enzyme at the heart of that network, glutamine‐dependent asparagine synthetase, which catalyses the transfer of an amino group from glutamine to aspartate to form asparagine and glutamate (Gaufichon, Reisdorf‐Crena, Rothstein, Chardona, & Suzuki, 2010; Xu et al., 2018).

The bread wheat (*Triticum aestivum*; genomes AABBDD) asparagine synthetase gene family comprises five genes per genome: *TaASN1*, *TaASN2*, *TaASN3.1*, *TaASN3.2* and *TaASN4* (Xu et al., 2018). Of these, *TaASN3* is the most highly expressed during early grain development, but *TaASN1* and *TaASN2* are the most highly expressed by mid‐development, with expression of *TaASN2* much higher than *TaASN1* (Curtis et al., 2019; Gao et al., 2016). Indeed, *TaASN2* expression in the grain at this stage is much higher than that of any of the other genes in any tissue, while its expression in other tissues is almost undetectable (Gao et al., 2016). Its expression in the embryo is much higher than in the endosperm (Curtis et al., 2019; Gao et al., 2016), with its expression in the embryo almost certainly the main determinant of free asparagine levels in the grain as a whole (Curtis et al., 2019). The A genome *TaASN2* gene has been shown to be much more highly expressed than the D genome version (Curtis et al., 2019), while the same study detected no expression of the B genome gene, consistent with the observation that the B genome gene is absent in some genotypes (Xu et al., 2018).

Here we review publicly available genome data for wheat and other cereals, including bread wheat (*T. aestivum*), its close relatives and more distantly related species such as maize (*Zea mays*), sorghum (*Sorghum bicolor*) and rice (*Oryza sativa*). Phylogenetic analyses were performed to show differences in the organisation of the gene family across the cereal species and a pathway constructed to describe how the cereal asparagine synthetase gene family has evolved.

## 2 IDENTIFYING AND COMPARING CEREAL ASPARAGINE SYNTHETASE GENES FROM PUBLICLY AVAILABLE GENOME DATA

The EnsemblPlants database (http://plants.ensembl.org/index.html) was used to provide genome information for each species, searching for asparagine synthetase genes based on annotation or by BLASTn or BLASTp searches (Altschul, Gish, Miller, Myers, & Lipman, 1990) using wheat asparagine synthetase gene nucleotide or derived amino acid sequences. The DNA sequences were then downloaded and viewed using Geneious (Geneious 10.1.3; Kearse et al., 2012), and nucleotide sequences upstream of the translation start codon and downstream of the translation stop codon were discarded. The nucleotide sequences were then aligned using Geneious Alignment on its default settings, with a cost matrix of 65% similarity using a global alignment with free end gaps, and the similarities between the genes were quantified using the nucleotide alignment matrix that was generated. All cDNA alignments were confirmed using MUSCLE alignments within the Geneious package (Kearse et al., 2012). Similarity values were generated between the asparagine synthetase genes of a single species and in comparison to the wheat genes.

The alignments were used to build trees, using Geneious tree builder software (Kearse et al., 2012), to visualise the relationship between the genes. The Jukes–Cantor model was used for genetic distance, and the tree was built via a neighbour‐joining method. The Jukes–Cantor model is a simple substitution model which assumes that all bases have the same equilibrium base frequency and that nucleotide substitutions occur at equal rates (Jukes & Cantor, 1969). The neighbour‐joining method was employed as it allowed for unrooted trees to be generated quickly without assuming a molecular clock (Saitou & Nei, 1987). A resampling method was also employed. Bootstrapping (Felsenstein, 1985) was performed with 100 replicates, creating consensus trees, which were then used to examine the relationship of the genes across the cereal species. The consensus tree allowed for an estimation of the support for each clade, and a 50% threshold was applied in a majority rule consensus tree. The length of the branches in the consensus tree corresponded to the average over all trees containing the clade, with the length of the tip branches calculated by averaging over all trees. The arabidopsis (*Arabidopsis thaliana*) gene, *AtASN1* (Lam, Peng, & Coruzzi, 1994), was used as an outgroup to anchor the trees. The scale bars on the tree represented the length of the branches and were expressed in units of substitutions per site of the sequence alignment.

## 3 THE ASPARAGINE SYNTHETASE GENE FAMILY OF THE TRITICEAE

### 3.1 Bread wheat (*T. aestivum*)

The Triticeae are a tribe within the Pooideae subfamily of the family Poaceae, comprising cultivated and wild wheat, rye and barley species. The asparagine synthetase gene family of bread wheat (*T. aestivum*; hexaploid, genomes AABBDD) has been described in some detail. It comprises five genes per genome (Gao et al., 2016; Xu et al., 2018), with single copies of *TaASN1* on chromosomes 5A, 5B and 5D, single copies of *TaASN2* on chromosomes 3A, 3B (missing in some varieties) and 3D, two copies of *TaASN3* (*TaASN3.1* and *TaASN3.2*) on chromosomes 1A, 1B and 1D and single copies of *TaASN4* on chromosomes 4A, 4B and 4D. These are annotated as *TaASN1–TaASN5* in some datasets, but the high sequence identity, similar intron/exon pattern and close chromosomal proximity show that *TaASN3.1* and *TaASN3.2* are paralogues resulting from a relatively recent gene duplication (Xu et al., 2018).

**FIGURE 1:**
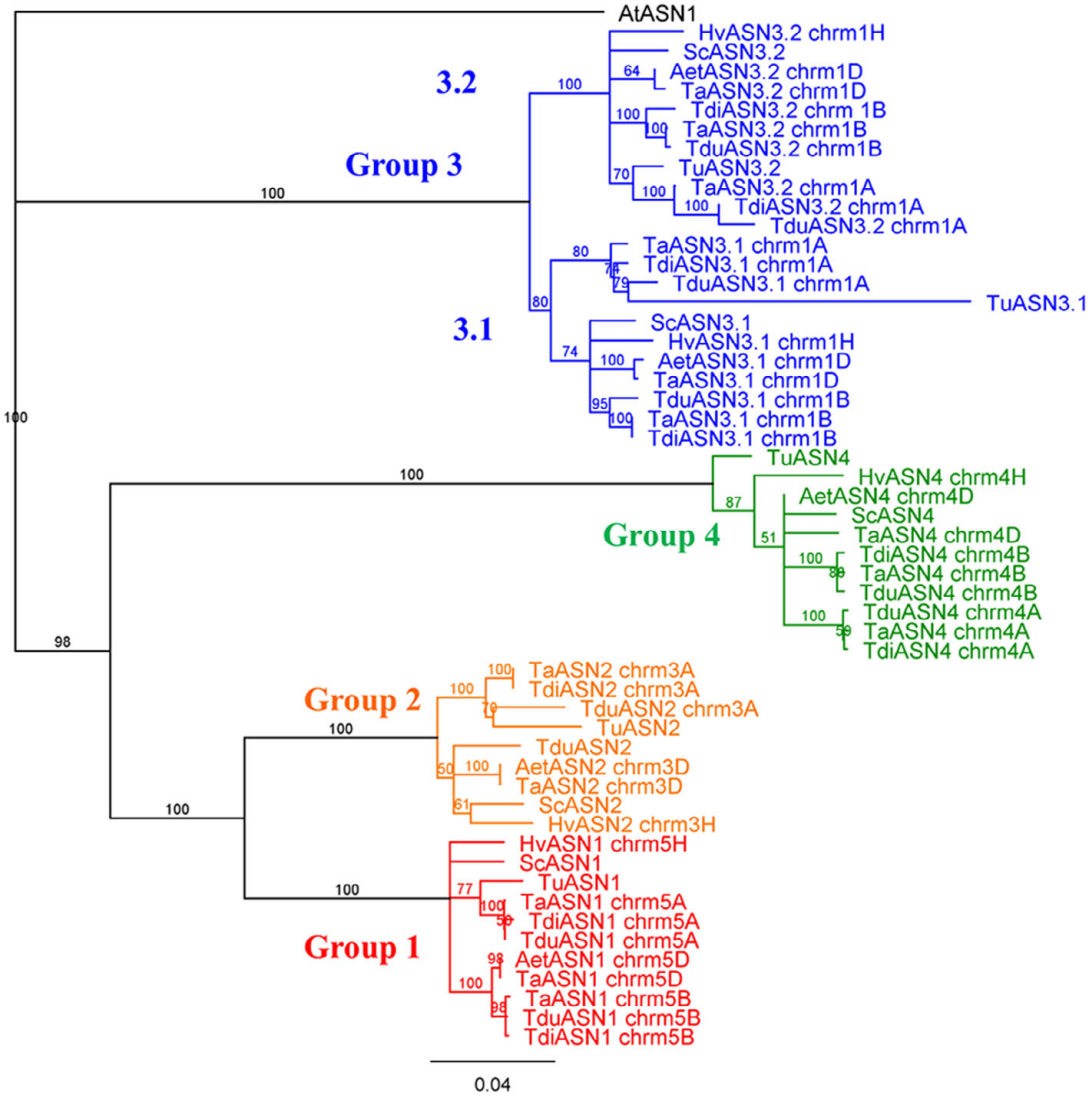
Phylogenetic tree of the asparagine synthetase genes in Triticeae species, including bread wheat (*Triticum aestivum*; genes shown with a Ta prefix), emmer wheat (*Triticum dicoccoides*; Tdi prefix), durum (pasta) wheat (*Triticum durum*; Tdu prefix), einkorn wheat (*Triticum urartu*; Tu prefix), Tausch's goatgrass (*Aegilops tauschii*; Aet prefix), barley (*Hordeum vulgare*; Hv prefix) and rye (*Secale cereale*; Sc prefix). Genes are listed in Tables 2, 3, 4 and S1. The branch labels show the consensus support in the clade from bootstrapping, as a percentage. The scale bar represents the length of the branches, expressed in units of substitutions per site of the sequence alignment. The arabidopsis (*Arabidopsis thaliana*) gene, *AtASN1*, was used as an outgroup to anchor the tree

A similarity matrix was generated using nucleotide sequence data for *TaASN1‐4* of variety Chinese Spring, available from the EnsemblPlants genome database (International Wheat Genome Sequencing Consortium (IWGSC), 2018, http://plants.ensembl.org/index.html). The matrix is shown in Tables 1 and S1 (Similarity Matrix 1.1 and subsequent matrices are all given in Supporting Information). This confirmed the relationships between the genes, with the two showing most similarity being *TaASN3.1* and *TaASN3.2* (92.1–93.0% nucleotide sequence identity). *TaASN1* and *TaASN2* also showed greater similarity to each other than to the other genes (84.4–84.6% identity). Within the groups of homeologues, *TaASN1* showed the highest similarity at 97.6–99.1% identity, while *TaASN2* showed 96.3%, *TaASN3.1* 95.4–96.6%, *TaASN3.2* 95.7–96.6% and *TaASN4* 95.1–97.6%. A phylogenetic tree was generated and is shown in Figure 1.

**TABLE 1:** Matrix of similarity calculated from a nucleotide alignment of the wheat asparagine synthetase genes

	*TaASN1* chrm5A	*TaASN1* chrm5B	*TaASN1* chrm5D	*TaASN2* chrm3A	*TaASN2* chrm3D	*TaASN3.1* chrm1A	*TaASN3.1* chrm1B	*TaASN3.1* chrm1D	*TaASN3.2* chrm1A	*TaASN3.2* chrm1B	*TaASN3.2* chrm1D	*TaASN4* chrm4A	*TaASN4* chrm4B	*TaASN4* chrm4D
*TaASN1* chrm5A		97.6	97.8	84.4	84.4	70.2	69.4	69.9	69.5	68.9	70.7	69.9	70.5	70.3
*TaASN1* chrm5B	97.6		99.1	84.5	84.5	70.1	69.6	70.1	69.5	69	70.7	69.6	70.2	70.1
*TaASN1* chrm5D	97.8	99.1		84.5	84.6	70.4	69.9	70.3	69.9	69.4	71	69.5	70.1	69.9
*TaASN2* chrm3A	84.4	84.5	84.5		96.3	69.2	69.3	69.3	68.7	68.1	69.8	67.9	69.3	69
*TaASN2* chrm3D	84.4	84.5	84.6	96.3		69.8	70	69.8	69.1	68.5	69.9	68.6	69.9	69.5
*TaASN3.1* chrm1A	70.2	70.1	70.4	69.2	69.8		96.4	95.4	92.1	92.1	92.7	62.2	62.6	62.8
*TaASN3.1* chrm1B	69.4	69.6	69.9	69.3	70	96.4		96.6	92.3	92	92.7	61.7	62.2	62.4
*TaASN3.1* chrm1D	69.9	70.1	70.3	69.3	69.8	95.4	96.6		92.5	93	93	61.9	62.3	62.6
*TaASN3.2* chrm1A	69.5	69.5	69.9	68.7	69.1	92.1	92.3	92.5		96.6	95.7	62.5	63	63.3
*TaASN3.2* chrm1B	68.9	69	69.4	68.1	68.5	92.1	92	93	96.6		95.7	61.8	62.6	62.8
*TaASN3.2* chrm1D	70.7	70.7	71	69.8	69.9	92.7	92.7	93	95.7	95.7		64	64.5	64.6
*TaASN4* chrm4A	69.9	69.6	69.5	67.9	68.6	62.2	61.7	61.9	62.5	61.8	64		95.1	95.8
*TaASN4* chrm4B	70.5	70.2	70.1	69.3	69.9	62.6	62.2	62.3	63	62.6	64.5	95.1		97.6
*TaASN4* chrm4D	70.3	70.1	69.9	69	69.5	62.8	62.4	62.6	63.3	62.8	64.6	95.8	97.6	

*Note*: Genes are listed in Tables 2 and S1. The values presented are the percentage of bases which are identical, expressed to one decimal place, and the colours are used to visualise the similarity between genes.

The analysis confirmed a previous observation (Xu et al., 2018) that variety Chinese Spring lacks a B genome *TaASN2* gene. Analysis of variety Spark and a doubled haploid line, SR3, from a Spark × Rialto mapping population also found no evidence of expression of a B genome *TaASN2* gene (Curtis et al., 2019), suggesting that the gene might be missing in those genotypes as well. However, the variety Cadenza genome data, also available from EnsemblPlants, do contain a B genome *TaASN2* gene, so the absence of the B genome *TaASN2* gene is not universal in modern bread wheat genotypes.

### 3.2 Emmer wheat (*T. dicoccoides*)

*Triticum dicoccoides*, also known as *Triticum turgidum* subsp. *dicoccoides*, and commonly as emmer or hulled wheat, is a tetraploid (genomes AABB). It is widely regarded as the wild progenitor of cultivated *Triticum durum* (pasta wheat) and *Triticum dicoccum* (also known as *T. turgidum* subsp. *durum* and *T. turgidum* subsp. *dicoccum*), the main difference between the wild and cultivated species being that the ripe seed heads of the wild wheat shatter to spread the seed, whereas the seed heads of the cultivated species do not. Emmer wheat is also considered to be one of the progenitors of bread wheat, with bread wheat forming from a hybridisation event between *T. dicoccoides* (AABB genomes) and *Aegilops tauschii* (DD) around 8,000 years ago (Matsuoka, 2011). Along with einkorn wheat (*Triticum urartu*), emmer wheat was one of the first crops to be domesticated and it was widely cultivated in the ancient world.

The *T. dicoccoides* accession used for the genome assembly was Zavitan, because of the availability of pre‐existing genetic data for this genotype (WEWSeq v.1.0, INSDC Assembly; Avni et al., 2017). Nine asparagine synthetase genes were identified in the database, of which genes with Ensembl reference numbers TRIDC5AG025640 and TRIDC5BG026790 were located on chromosomes 5A and 5B, respectively, TRIDC3AG009140 was located on chromosome 3A, with no homeologue on chromosome 3B, TRIDC4AG015200 and TRIDC4BG033760 were located on chromosomes 4A and 4B, respectively, while TRIDC1AG056280 and TRIDC1AG062090 genes were located on chromosome 1A, with TRIDC1BG064720 and TRIDC1BG071210 on chromosome 1B.

These genes fall into the same groups as the *TaASN* genes from the A and B genomes (Table S1, Similarity Matrix 1.2), and this was confirmed by phylogenetic analysis (Figure 1). We therefore annotate TRIDC5AG025640 and TRIDC5BG026790 as *TdiASN1*, TRIDC3AG009140 as *TdiASN2*, TRIDC1AG056280 and TRIDC1BG064720 as *TdiASN3.1*, TRIDC1AG062090 and TRIDC1BG071210 as *TdiASN3.2* and TRIDC4AG015200 and TRIDC4BG033760 as *TdiASN4* (Table 2; using the Tdi prefix to distinguish the *T. dicoccoides* genes from the *T. durum* genes described below). The fact that the B genome *TdiASN2* gene is missing is somewhat unexpected given that, as stated above, some hexaploid wheat varieties do have this gene.

**TABLE 2:** Asparagine synthetase gene families of different species of wheat (*Triticum* and *Aegilops* spp.); all members of the Triticeae tribe of the Pooideae subfamily.

Group	Bread wheat *Triticum aestivum* (AABBDD)			Emmer wheat *Triticum dicoccoides* (AABB)			Durum (pasta) wheat *Triticum durum* (AABB)		
	Gene name	Chromosome	Ensembl reference	Gene name	Chromosome	Reference	Gene name	Chromosome	Reference
1	*TaASN1*	5A	TraesCS5A02G153900	*TdiASN1*	5A	TRIDC5AG025640	*TduASN1*	5A	TRITD5Av1G114980
		5B	TraesCS5B02G152600		5B	TRIDC5BG026790		5B	TRITD5Bv1G097460
		5D	TraesCS5D02G159100						
2	*TaASN2*	3A	TraesCS3A02G077100	*TdiASN2*	3A	TRIDC3AG009140	*TduASN2*	3A	TRITD3Av1G021350
		3B			3B			3B	TRITD0Uv1G024260
		3D	TraesCS3D02G077300						
3.1	*TaASN3.1*	1A	TraesCS1A02G382800	*TdiASN3.1*	1A	TRIDC1AG056280	*TduASN3.1*	1A	TRITD1Av1G215640
		1B	TraesCS1B02G408200		1B	TRIDC1BG064720		1B	TRITD1Av1G222870
		1D	TraesCS1D02G390500						
3.2	*TaASN3.2*	1A	TraesCS1A02G422100	*TdiASN3.2*	1A	TRIDC1AG062090	*TduASN3.2*	1A	TRITD1Bv1G207110
		1B	TraesCS1B02G453600		1B	TRIDC1BG071210		1B	TRITD1Bv1G220260
		1D	TraesCS1D02G430300						
4	*TaASN4*	4A	TraesCS4A02G109900	*TdiASN4*	4A	TRIDC4AG015200	*TduASN4*	4A	TRITD4Av1G053230
		4B	TraesCS4B02G194400		4B	TRIDC4BG033760		4B	TRITD4Bv1G119970
		4D	TraesCS4D02G195100						

Group	Einkorn wheat *Triticum urartu* (AA)			Goat grass *Aegilops tauschii* (DD)					
	Gene name	Chromosome	Reference	Gene name	Chromosome	Reference			
1	*TuASN1*	ND	TRIUR3_11865	*AetASN1*	5	AET5Gv20393100			
2	*TuASN2*	ND	TRIUR3_15772	*AetASN2*	3	AET3Gv20170100			
3.1	*TuASN3.1*	ND	TRIUR3_27580	*AetASN3.1*	1	AET1Gv20919800			
3.2	*TuASN3.2*	ND	TRIUR3_05036	*AetASN3.2*	1	AET1Gv21000200			
4	*TuASN4*	ND	TRIUR3_21196	*AetASN4*	4	AET4Gv20505300			

Abbreviations: ND, not determined.

When the bread wheat genes were added into the alignment matrix (Table S1, Similarity Matrix 1.3), the shared identity reflected the close evolutionary history between the two species and the evolutionary constraints on asparagine synthetase function. For example, there was 100% identity between *TdiASN2* (TRIDC3AG009140) and the chromosome 3A version of *TaASN2*, and 96.5% identity with the chromosome 3D *TaASN2*.

There were also high similarities between the *ASN1* genes on chromosome 5, the A genome *TdiASN1* (TRIDC5AG025640) showing 99.5% identity with the A genome *TaASN1*, and the B genome *TdiASN1* (TRIDC5BG026790) showing 99.7% identity with the B genome *TaASN1*. The chromosome 5A and 5B *TdiASN1* genes shared 97.3 and 99.2% identity, respectively, with the chromosome 5D *TaASN1*, and 84.3–84.4% identity with the *TaASN2* (3A and 3D) genes. The reciprocal pairing of *TdiASN2* (TRIDC3AG009140) with the *TaASN1* genes showed 83.8–84.1% identity, again confirming the relatively close relationship between the asparagine synthetase genes from the two species.

Comparing the two *TdiASN3.1* and *TdiASN3.2* genes on chromosomes 1A and 1B of *T. dicoccoides* with their *TaASN3* counterparts, 99.5% identity could be seen between *TdiASN3.1* (TRIDC1AG056280) and the A genome *TaASN3.1*, and 100% identity between *TdiASN3.1* (TRIDC1BG064720) and the B genome *TaASN3.1*. Predictably, both *TdiASN3.1* genes showed a lower similarity to the *TaASN3.1* gene from chromosome 1D. For the *TdiASN3.2* and *TaASN3.2* genes, the chromosome 1A pair (including TRIDC1AG062090) showed 99.6% identity whereas the 1B pair (including TRIDC1BG071210) shared only 98.1% identity, with both *TdiASN3.2* genes showing a lower similarity to the D genome homeologue of *TaASN3.2*.

The two *TdiASN4* genes also showed very high similarity with their *T. aestivum* counterparts, with the A genome gene (TRIDC4AG015200) showing 99.2% identity with the A genome *TaASN4* gene and the B genome gene (TRIDC4BG033760) showing 99.6% identity with the B genome *TaASN4* gene. Both showed between 95.9 and 97.7% identity with the *TaASN4* genes from different genomes, and they showed 96.6% identity with each other.

### 3.3 Pasta wheat (*T. durum*)

Cultivated pasta wheat (*T. durum*; also known as *T. turgidum* subsp. *durum*) is also a tetraploid (genomes AABB) and believed to be a direct descendent of domesticated emmer wheat (Peng, Sun, & Nevo, 2011). It is widely grown in the Middle East and southern Europe (Tedone, Ali, & de Mastro, 2017). The variety Svevo was used for the genome assembly (Svevo.v1, INSDC Assembly GCA_900231445; Maccaferri et al., 2019), with the Svevo × Zavitan genetic map used to order and orient the scaffolds.

Curci et al. (2018) characterised two asparagine synthetase genes in *T. durum*, but in our analysis 10 genes were identified in the Svevo genome (Table 2), all except TRITD0Uv1G024260 of which were already annotated as encoding asparagine synthetases. TRITD5Av1G114980 and TRITD5Bv1G097460 were located on chromosome 5A and 5B, respectively, and shared 97.4% similarity (Table S1, Similarity Matrix 1.4); TRITD4Av1G053230 and TRITD4Bv1G119970 were located on chromosomes 4A and 4B, sharing 92.9% similarity; TRITD1Av1G215640 and TRITD1Av1G222870 were both located on chromosome 1A, showing 93.5% similarity; while TRITD1Bv1G207110 and TRITD1Bv1G220260 were located on chromosome 1B, showing 92.9% similarity. TRITD3Av1G021350 was located on chromosome 3A and while none of the genes was assigned to chromosome 3B it is likely that an unassigned gene, TRITD0Uv1G024260, represented the chromosome 3B homeologue as it showed 91.0% similarity to TRIT3Av1g021350.

When aligned to the bread wheat genes (Table S1, Similarity Matrix 1.5), TRITD5Av1G114980 and TRITD5Bv1G097460 showed 99.8% similarity with the chromosome 5A and 5B versions, respectively, of the *TaASN1* genes. TRITD3Av1G021350 and TRITD0Uv1G024260 showed most similarity to the *TaASN2* genes, with TRITD0Uv1G024260 showing slightly more similarity to the A genome *TaASN2* than TRITD3Av1G021350 did (95.6% vs. 94.7%), despite TRITD3Av1G021350 being assigned to chromosome 3A and unassigned gene TRITD0Uv1G024260 presumably being from chromosome 3B. Obviously there was no B genome *TaASN2* gene to compare these genes with.

Of the two genes on each of chromosomes 1A and 1B, TRITD1Av1G215640 showed 94.3–98.2% similarity to the *TaASN3.1* genes and 90.5–91.4% to the *TaASN3.2* genes, with the greatest similarity seen with the chromosome 1A version of *TaASN3.1*. TRITD1Bv1G207110 showed 94.5–97.7% similarity to the *TaASN3.1* genes, with the highest similarity seen to the 1B homeoallele, and 92.5–94.2% similarity to the *TaASN3.2* genes. TRITD1Av1G222870 showed the highest similarity, 93.2–97.1%, with the highest value coming from comparison with *TaASN3.2* on chromosome 1A. TRITD1Bv1G220260 showed 95.7–98.8% to the *TaASN3.2* genes and 92.3–92.9% to the *TaASN3.1* genes.

As such, we annotated TRITD5Av1G114980 and TRITD5Bv1G097460 as *TduASN1* 5A and 5B, respectively; TRITD4Av1G053230 and TRITD4Bv1G119970 as *TduASN4* 4A and 4B, respectively; with TRITD3Av1G021350 and TRITD0Uv1G024260 being annotated as *TduASN2* 3A and 3B, respectively. We annotated TRITD1Av1G215640 and TRITD1Av1G222870 as *TduASN3.1* 1A and 1B, with TRITD1Bv1G207110 and TRITD1Bv1G220260 as *TduASN3.2* 1A and 1B.

When comparing the *T. dicoccoides*, *T. durum* and *T. aestivum* genes (Table S1, Similarity Matrix 1.6), all the orthologues showed over 96% similarity with each other, reflecting their close evolutionary history, with the lowest level of similarity seen between the chromosome 4A *ASN4* genes (96.2%). The *TdiASN1* genes showed 99.7–99.9% similarity to their respective orthologues in *T. durum*, and 99.5–99.8% similarity to the *TaASN1* 5A and 5B genes, with the 5D gene showing 97.3–99.3% similarity. The *TdiASN2* gene, located on chromosome 3A, was identical to the *TaASN2* 3A gene but only 95.0% similar to the *TduASN2* 3A gene, with the *TduASN2* and *TaASN2* genes also showing 95.0% similarity.

For the *ASN3* genes, the *TdiASN3.1* genes showed higher similarity to the *TaASN3.1* genes (95.6–100%) than the *TduASN3.1* genes did (94.4–98.5%). The same was true for the *ASN3.2* genes, where *TdiASN3.1* showed similarities from 95.4–99.6% to the *TaASN3.2* genes compared to the 92.8–98.4% between the *TaASN3.2* and *TduASN3.2* genes. The *T. dicoccoides* and *T. durum* genes showed a similarity of 94.5–98.7% and 94–99.6% for the *ASN3.1* and *ASN3.2* genes, respectively. The *TaASN4* genes were also more similar to the *TdiASN4* genes (96.0–99.7%) than to the *TduASN4* genes (92.9–99.9%). Interestingly, the 4A *TdiASN4* gene showed a marginally higher similarity to the 4B version of the *TduASN4* genes (96.6% compared to 99.7%).

### 3.4 Goat grass (*Aegilops tauschii*)

*Aegilops tauschii*, commonly called Tausch's goat grass or rough‐spike hard grass, is the diploid progenitor of the D genome of bread wheat (McFadden & Sears, 1946). The genome assembly uses the subspecies *A. tauschii* subsp. *strangulata* (Aet v4.0, INSDC Assembly; Luo et al., 2017).

The *A. tauschii* asparagine synthetase gene family showed the same organisation as those of *T. aestivum*, *T. dicoccoides* and *T. durum*, with single genes on chromosomes 3, 4 and 5, and two genes on chromosome 1 (Tables 2 and S1 [Similarity Matrix 1.7] and Figure 1). The gene on chromosome 5, AET5Gv20393100, showed 100% identity with the *TaASN1* gene on chromosome 5D of *T. aestivum*, while the gene on chromosome 3, AET3Gv20170100, was identical to the to the *TaASN2* gene on chromosome 3D of *T. aestivum* (Table S1, Similarity Matrix 1.8). These genes can therefore be annotated as *AetASN1* and *AetASN2*, respectively (Table 2).

AET1Gv21000200 and AET1Gv20919800 on chromosome 1 showed 92.7% identity with each other, suggesting that they are paralogues like *TaASN3.1* and *TaASN3.2*, with AET1Gv20919800 showing greater identity to *TaASN3.1* (99.6% for the D genome *TaASN3.1* compared with 92.9% for the D genome *TaASN3.2*), whereas AET1Gv21000200 showed 97.4% identity with the D genome *TaASN3.2* gene compared with less than 93% identity to the D genome *TaASN3.1* gene. AET1Gv20919800 and AET1Gv21000200 can therefore be annotated as *AetASN3.1* and *AetASN3.2*, respectively. Similarly, Aet20505300 on chromosome 4 showed highest identity to the *TaASN4* gene, at 97.3–99.9%, with highest similarity to the D genome *TaASN4*.

Analysis of data from an RNA‐seq analysis of the leaves of seedlings of 10 *A. tauschii* accessions (Nishijima, Yoshida, Motoi, Sato, & Takumi, 2016) showed considerable variation in asparagine synthetase gene expression between different genotypes, with *AetASN1* the most highly expressed in most. *AetASN2* expression was not detectable, consistent with *AetASN2* being expressed only in the grain, in similar fashion to *TaASN2* of bread wheat (Curtis et al., 2019; Gao et al., 2016), but no data are available yet to confirm this.

### 3.5 Einkorn wheat (*T. urartu*)

*Triticum urartu*, commonly known as red wild einkorn (German einkorn, meaning single grain) wheat, is the diploid progenitor of the A genome of cultivated wheat (Dvořák, Terlizzi, Zhang, & Resta, 1993), and its genome was analysed initially to provide information on the evolution of hexaploid bread wheat (*T. aestivum*) and tetraploid durum wheat (*T. durum*). The accession G1812 was used for the genome analysis, but to date no chromosomes have been assembled from the data, with scaffolds presented instead (ASM34745v1, INSDC Assembly; Ling et al., 2013), so chromosomal positioning information was unavailable.

Initial assessment of the asparagine synthetase genes showed a surprisingly high number of 8 (Table S1, Similarity Matrix 1.9). These were: TRIUR3_05036, TRIUR3_11865, TRIUR3_15772, TRIUR3_21196, TRIUR3_27580, TRIUR3_27838, TRIUR3_33442 and TRIUR3_34781. Within the group, the highest identity was seen between TRIUR3_11865 and TRIUR3_15772 at 80.8%, with TRIUR3_27580 and TRIUR3_05036 (73.2%) and TRIUR3_33442 and TRIUR3_34781 (73.5%) showing high similarities as well.

Comparison of the eight genes with the bread wheat genes showed TRIUR3_27838, TRIUR3_33442 and TRIUR3_34781 to be much more divergent than any of the asparagine synthetase genes from other wheat species (Table S1, Similarity Matrix 1.10). These three genes were found to contain no introns, and wider BLAST searches showed them to be of bacterial origin, with TRIUR3_27838 showing high identity to an asparagine synthetase B gene from *Sphingobacterium* sp. 2c‐3 (Genbank WP_116774391.1), while TRIUR3_34781 and TRIUR3_33442 showed 100% identity with asparagine synthetase B genes from *Frankliniella occidentalis*, a bacterial symbiont, BFo1, of western flower thrips (Genbank KYP89277.1, KMV69586.1 for TRIUR3_34781; KMV69586.1 for TRIUR3_33442). These three gene sequences therefore clearly result from contamination of the DNA used in the genome analysis and were not considered further.

Of the others, TRIUR3_11865 showed very high levels of similarity to *TaASN1* (93.3–95.6%), TRIUR3_15772 to *TaASN2* (90.9–93.4%), TRIUR3_21196 to *TaASN4* (88.4–89.9%) and TRIUR3_05036 to *TaASN3.2* (90.2–91.8%; Table 2 and Figure 1). That leaves TRIUR3_27580 as the *TaASN3.1* homologue; it only showed 81.6–84.6% identity with *TaASN3.1*, which was lower than expected, but it did cluster with *TaASN3.1*, *TdiASN3.1* and *TduASN3.1* in the phylogenetic analysis (Figure 1).

### 3.6 Barley (*Hordeum vulgare*)

Barley was domesticated alongside wheat in the Fertile Crescent over 10,000 years ago (Badr et al., 2000). It is a diploid close relative of wheat and, like wheat, its haploid genome comprises seven chromosomes. Variety Morex was used for the genome assembly (IBSC v2, INSDC Assembly; International Barley Genome Sequencing Consortium; Beier et al., 2017).

Five *HvASN* genes were identified from the genome data (Table 3 and Figure 1): HORVU3Hr1G013910 on chromosome 3 (which has been annotated as an *ASN2* gene, https://www.uniprot.org/uniprot/Q84LA5), HORVU4Hr1G056240 on chromosome 4, HORVU1Hr1G084370 and HORVU1Hr1G092110 on chromosome 1 and HORVU5Hr1G048100 on chromosome 5. Thus, the barley gene family has the same basic organisation as the wheat gene family, with a single copy of each gene. The genes on chromosome 1 showed the highest similarity to each other, with 92.6% identity (Table S1, Similarity Matrix 1.11). This was followed by 80.1% identity between HORVU3Hr1G013910 and HORVU5Hr1G048100, with the lowest similarity values shown between HORVU4Hr1G056240 and the two genes located on chromosome 1.

**TABLE 3:** Asparagine synthetase gene families of barley (*Hordeum vulgare*) and rye (*Secale cereale*), of the Triticeae, and brachypodium (*Brachypodium distachyon*) of the Brachypodiae (a separate tribe within the Pooideae)

Group	Barley (*H. vulgare*)			Rye (*S. cereale*)			Brachypodium (*B. dystachion*)		
	Gene name	Chromosome	Reference	Gene name	Chromosome	Scaffold	Gene name	Chromosome	Reference
1	*HvASN1*	5	HORVU5Hr1G048100	*ScASN1*	ND	370516	*BdASN1*	4	BRADI_4g45010v3
2	*HvASN2*	3	HORVU3Hr1G013910	*ScASN2*	ND	174491			
3.1	*HvASN3*	1	HORVU1Hr1G084370	*ScASN3.1*	ND	3445	*BdASN3*	2	BRADI_2g21050v3
3.2	*HvASN4*	1	HORVU1Hr1G092110	*ScASN3.2*	ND	1245, 81708, 36651			
4	*HvASN5*	4	HORVU4Hr1G056240	*ScASN4*	ND	Scaffold 527072	*BdASN4*	1	BRADI_1g65540v3

*Note*: The barley genes previously annotated as *HvASN4* and *HvASN5* (Avila‐Ospina, Marmagne, Talbotec, & Krupinska, 2015) are assigned to groups 3.2 and 4, respectively.

Abbreviations: ND, not determined.

The *HvASN* genes were aligned to and compared with the *TaASN* genes (Table S1 [Similarity Matrix 1.12] and Figure 1). *HvASN2* (HORVU3Hr1G013910) showed 91.8–92.3% identity to *TaASN2*, followed by 81–81.2% identity to the *TaASN1* gene, while HORVU5Hr1G048100 showed 96.3–96.5% identity to the *TaASN1* genes, with 83.9% identity to the *TaASN2* genes. High levels of identity were seen between the barley and wheat *ASN3* genes (92.0–96.8%), with HORVU1Hr1G084370 showing higher identity to *TaASN3.1* than to *TaASN3.2* (94.9–96.1% versus 92.0–92.4%) and HORVU1Hr1G092110 being more similar to *TaASN3.2* than *TaASN3.1* (94.5–96.8% compared with 92.3–92.8%). HORVU4Hr1G056240 on chromosome 4 was most similar to *TaASN4s* (94.6–95.3% identity).

Møller, Taylor, Rasmussen, and Holm (2003) initially described two asparagine synthetase genes in barley, but Avila‐Ospina et al. (2015) identified five, although they annotated *HvASN3.2* as *HvASN4* and *HvASN4* as *HvASN5*. Gene expression data for germinating barley (Zhang et al., 2016) and barley seedlings (International Barley Genome Sequencing Consortium, 2012) show *HvASN3* and *HvASN4* to be the most highly expressed, with very low levels of expression for *HvASN2*. Again, this is consistent with *HvASN2* being expressed grain‐specifically, but to date there are no data to support this.

### 3.7 Rye (*Secale cereale*)

Rye is a diploid member of the Triticeae which diverged from the ancestors of wheat relatively recently. Genome data were obtained through the whole‐genome sequencing of the winter rye inbred line, Lo7 (Bauer et al., 2017). The data in that resource are presented as scaffolds, with no chromosome assemblies. Five asparagine synthetase genes were identified, one each on scaffolds 370516, 174491, 3445 and 527072, with a fifth located across three scaffolds (1245, 81708 and 36651).

From the initial BLAST search, the scaffold 370516 gene was annotated as *ScASN1*, the scaffold 174491 gene as *ScASN2*, the scaffold 3445 and the tri‐scaffold gene as *ScASN3* and the scaffold 527072 gene as *ScASN4*. When aligned with each other (Table S1, Matrix 1.13), the highest similarity was seen between the two *ScASN3* genes, at 92.6%. This was followed by the *ScASN1* and *ScASN2* genes at 83.5% similarity, with the *ScASN4* gene showing the lowest similarity to the other genes at 63.5–69.3%.

The annotations were confirmed when the bread wheat genes were added to the alignment matrix (Table S1, Matrix 1.14). *ScASN1* showed 97.3–98.1% similarity to the *TaASN1* genes while *ScASN2* showed 95.1 and 96.1% similarity to the 3A and 3D *TaASN2* genes, respectively. The scaffold 3445 *ScASN3* gene was identified as *ScASN3.1*, showing 95.9–97.6% similarity to the *TaASN3.1* genes and 92.4–92.9% to the *TaASN3.2* genes, while the tri‐scaffold gene showed 95.5–97.7% similarity to the *TaASN3.2* genes but only 92.5–93.1% similarity to the *TaASN3.1* genes. The *ScASN4* gene showed 94.9–97.0% similarity to the *TaASN4* genes. The *ScASN* genes differed in which of the A, B or D genome versions of the *TaASN* genes they were most similar to, although the differences were generally small. The *ScASN1* showed the highest similarity to the A genome of *TaASN1*, while *ScASN2*, *ScASN3.1* and *ScASN4* were most similar to the D genome versions of the corresponding *TaASN* genes, and *ScASN3.2* was most similar to the B genome version of *TaASN3.2*.

### 3.8 Comparison and phylogenetic analysis of all the Triticeae genes

A similarity matrix was constructed for all of the genes from the Triticeae species (Table S1, Matrix 1.15) and a phylogenetic tree of all the genes was constructed (Figure 1). This confirmed that the asparagine synthetase gene families were the same in all the species, with five genes per genome, assigned to four groups.

## 4 BRACHYPODIUM (*BRACHYPODIUM DISTACHYON*)

Brachypodium is a model species for the cereals, and particularly the Pooideae subfamily, although it is usually classified in its own tribe of Brachypodiae. Its common name is purple false brome, but in science circles nowadays it is commonly just called brachypodium and we will continue to use that name. It is a diploid, with a small genome of approximately 355 megabases and a short life cycle (Bennett & Leitch, 2005; Draper et al., 2001), hence its suitability as a model species. It is closely related to the Triticeae but possesses only five chromosomes instead of seven. The Bd21 strain was used for the Ensembl genome, produced by the Joint Genome Institute (International Brachypodium Initiative, 2010, https://phytozome.jgi.doe.gov/pz/portal.html#!info?alias=Org_Bdistachyon).

Three *BdASN* genes were identified (Table 3): BRADI_1g65540v3 on chromosome 1, BRADI_2g21050v3 on chromosome 2 and BRADI_4g45010v3 on chromosome 4. The three genes showed relatively little similarity to each other (Table S1, Similarity Matrix 2.1), with the highest identity seen between BRADI_1g65540v3 and BRADI_4g45010v3 at 70.8%. BRADI_4g45010v3 showed higher identity to BRADI_2g21050v3 and BRADI_1g65540v3 than the chromosome 1 and chromosome 2 genes did to each other.

Alignment of the brachypodium genes with the bread wheat genes (Table S1, Similarity Matrix 2.2) showed the chromosome 1 *BdASN* gene, BRADI_1g65540v3, to have highest similarity with *TaASN4*, with identity ranging from 86.9–87.8%. The chromosome 2 *BdASN* gene, BRADI_2g21050v3, showed highest similarity with the *TaASN3s*, with identities of 89.6–90.2% for *TaASN3.1* and 89.1–89.8% for *TaASN3.2*. The chromosome 4 gene, BRADI_4g45010v3, showed highest similarity to the *TaASN1s*, with identity ranging from 90.7–91.0%. Not surprisingly given the relatively high similarity between *TaASN1* and *TaASN2*, it also showed 84.4 and 84.7% identity, respectively, with the A and D genome *TaASN2* genes.

**FIGURE 2:**
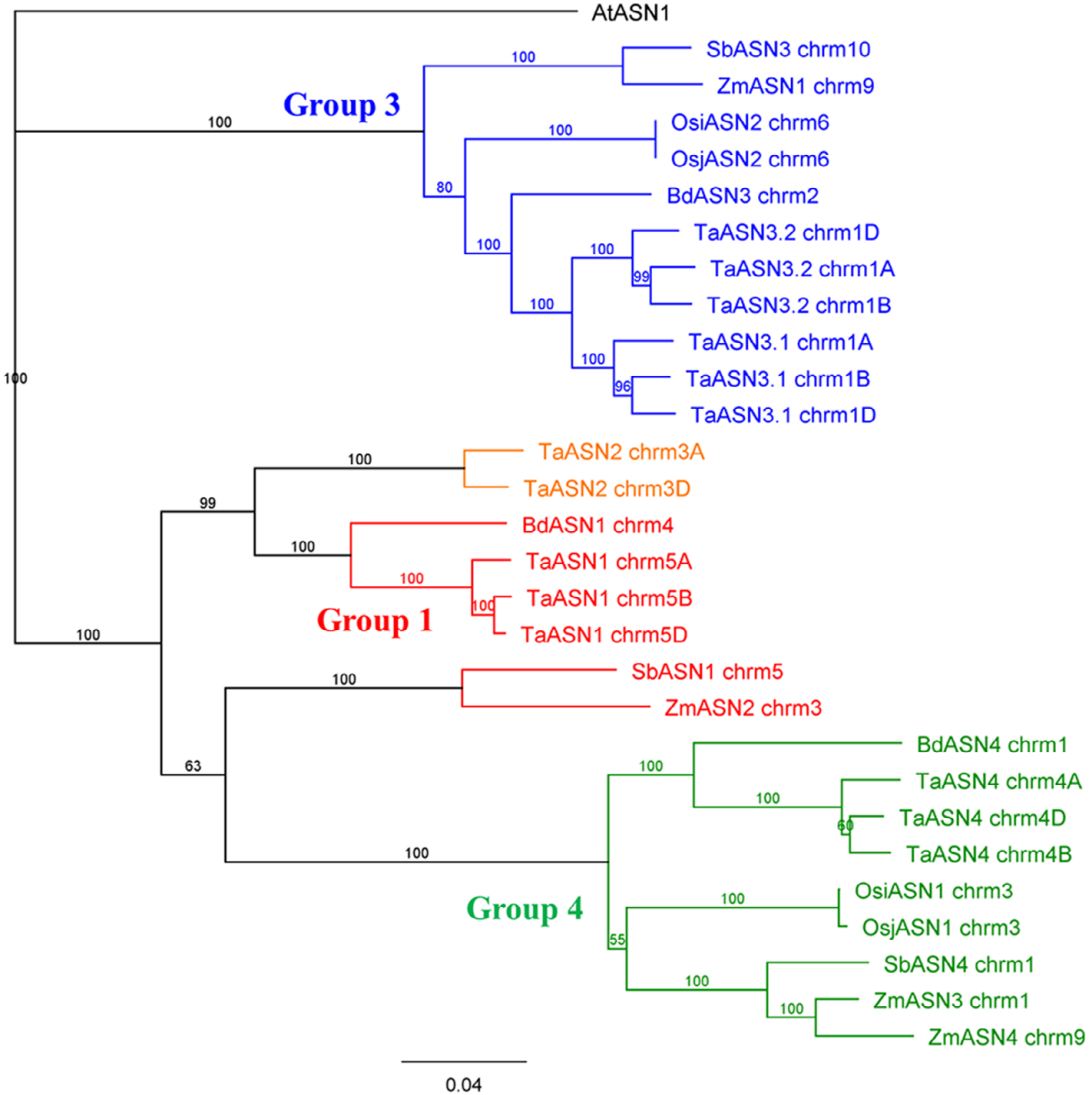
Phylogenetic tree of the asparagine synthetase genes in the Panicoideae species, maize (*Zea mays*, Zm prefix) and sorghum (*Sorghum bicolor*; Sb prefix), as well as brachypodium (*Brachypodium distachyon*; Bd prefix), rice (*Oryza sativa*; Os prefix) and wheat (*Triticum aestivum*; Ta prefix). Genes are listed in Tables 2, 3, 4 and S1. The branch labels show the consensus support in the clade from bootstrapping, shown as a percentage. The scale bar represents the length of the branches, expressed in units of substitutions per site of the sequence alignment. The arabidopsis (*Arabidopsis thaliana*) gene, *AtASN1*, was used as an outgroup to anchor the tree. Note that the maize and sorghum group 1 genes actually cluster with the group 4 genes, but are closely related to both groups and are assigned to group 1 based on similarity matrices (Table S1, Similarity Matrices 2.4 and 2.6)

The brachypodium data suggest that the relatively recent gene duplication event that gave rise to separate *ASN1* and *ASN2* genes, and the even more recent event that gave rise to the *ASN3.1* and *ASN3.2* genes did not occur in that species, and therefore presumably occurred in the Triticeae after brachypodium diverged from that line. This was confirmed by phylogenetic analysis (Figure 2). Brachypodium therefore has *BdASN1*, *BdASN3* and *BdASN4* genes (Table 3 and Figure 2).

Transcriptomic data for developing brachypodium grain (Davidson et al., 2012) shows *BdASN3* to be the most highly expressed asparagine synthetase gene during early development, with *BdASN1* the most highly expressed in the embryo by mid‐development, with expression in the embryo much higher than in the endosperm. This pattern of expression is similar to that of bread wheat (Curtis et al., 2019; Gao et al., 2016) with the exception, of course, that the most highly expressed gene in wheat grain, *TaASN2*, does not have an equivalent in brachypodium.

## 5 THE PANICOIDEAE: SORGHUM AND MAIZE

Sorghum (*S. bicolor*) is a widely grown cereal crop, able to grow under harsher conditions than most other cereal crops, including its close relative, maize (*Z. mays*). Its grain is used to make flat breads that form the staple food of many cultures, and it can also be used to make bioethanol. It is diploid, with a haploid chromosome number of 10.

The sorghum genome data utilised were from the Sorghum_bicolor_NCBIv3 assembly of the variety BTx623, generated by the Joint Genome Institute (Sorghum_bicolor_NCBIv3, INSDC Assembly GCA_000003195.3, April 2017; accessed on Emsembl; McCormick et al., 2018). Three asparagine synthetase genes were identified, one on chromosome 1, SORBI_3001G406800; one on chromosome 5, SORBI_3005G003200; and one on chromosome 10, SORBI_3010G110000 (Table 4). Within the group, SORBI_3001G406800 and SORBI_3005G003200 showed the highest nucleotide sequence identity at 70.9%, with less than 65% identity seen between SORBI_3010G110000 and the other two genes (Table S1, Similarity Matrix 2.3). Comparing them to the wheat asparagine synthetase genes (Table S1, Similarity Matrix 2.4), SORBI_3001G406800 on chromosome 1 showed the highest nucleotide sequence identity to *TaASN4* (83.1–84.1%), while SORBI_3005G003200 on chromosome 5 showed the highest identity with *TaASN1* and *TaASN2* (77.4–77.8% and 75.7–75.8%, respectively). SORBI_3010G110000 on chromosome 10 showed the highest similarity to the *TaASN3*s, with 83.2–84.1% nucleotide sequence identity to *TaASN3.1*, and 83.3–84.6% identity to *TaASN3.2*. We conclude that, as with brachypodium, the gene duplication events that led to *ASN1* and *ASN2* becoming two genes, and to the creation of two copies of *ASN3* did not occur in the Panicoideae (Figure 2). The analysis also showed the sorghum group 1 gene, *SbASN1*, to have diverged from the group 4 genes much less than the brachypodium and Triticeae group 1 and 2 genes; indeed, the phylogenetic analysis placed it marginally in the group 4 cluster (Figure 2), although we continue to regard it as a group 1 gene based on the similarity matrix.

**TABLE 4:** Asparagine synthetase gene families of maize (*Zea mays*) and sorghum (*Sorghum bicolor*) of the Panicoideae, and rice (*Oryza sativa*) of the Ehrhartoideae (also known as Oryzoideae)

Group	Sorghum (*S. bicolor*)			Maize (*Z. mays*)			Rice (*O. sativa*)		
	Gene name	Chromosome	Reference	Gene name	Chromosome	Reference	Gene name	Chromosome	Reference
1	*SbASN1*	5	SORBI_3005G003200	*ZmASN2*	3	Zm00001d044608			
3	*SbASN3*	10	SORBI_3010G110000	*ZmASN1*	9	Zm00001d045675	*OsASN2*	6 (*Indica*)	BGIOSGA021489
								6 (*Japonica*)	Os06g0265000
4	*SbASN4*	1	SORBI_3001G406800	*ZmASN3*	1	Zm00001d028750	*OsASN1*	3 (*Indica*)	BGIOSGA010942
				*ZmASN4*	9	Zm00001d047736		3 (*Japonica*)	Os03g0291500

*Note*: The maize genes are annotated *ZmASN1* to *ZmASN4* as previously (Todd et al., 2008), but are assigned to groups based on those identified in the other species. Maize also contains a number of partial genes that are not included. The rice genes, which have been annotated previously as *OsASN1* and *OsASN2* (Sakai et al., 2013), are assigned to groups 4 and 3, respectively.

A similar gene family was identified in maize (*Z. mays*). Maize was domesticated from wild teosinte (*Z. mays* ssp. *parviglumis*) in Central America around 9,000 years ago (Kistler et al., 2018). It has a large genome of approximately 2.4 gigabases, with a haploid chromosome number of 10 (Schnable et al., 2009). The reference genome is based on the B73 cultivar (B73 RefGen_v4, INSDC Assembly; Gramene; Jiao et al., 2017).

Four asparagine synthetase genes have previously been identified in maize (Todd et al., 2008) and this was confirmed, with Zm00001d028750 on chromosome 1, Zm00001d044608 on chromosome 3 and two genes, Zm00001d045675 and Zm00001d047736, on chromosome 9 (Table 4). Three additional partial genes were identified: Zm00001d010355 on chromosome 8 and Zm00001d031563 together with Zm00001d028766 on chromosome 1. Within the full‐length group, Zm00001d028750 and Zm00001d047736 were found to share 86.7% nucleotide sequence identity, whilst Zm00001d045675 and Zm00001d044608 only shared 56.3% (Table S1, Similarity Matrix 2.5). The three partial genes showed the highest similarity (80.5–83.4%) to Zm00001d044608.

Zm00001d045675 corresponds to a gene previously annotated as *ZmASN1*, Zm00001d044608 to a gene annotated as *ZmASN2*, Zm00001d028750 to *ZmASN3* and Zm00001d047736 to *ZmASN4* (Todd et al., 2008), while the three truncated genes are annotated in the database as *ZmASN1* homologues. However, comparing these genes to the wheat gene family (Figure 2 and Table S1 [Similarity Matrix 2.6]), both *ZmASN3* (Zm00001d028750) and *ZmASN4* (Zm00001d047736) showed greatest similarity to the *TaASN4s* (85.0–85.5% for *ZmASN3* and 76.1–76.3% for *ZmASN4*). These two genes presumably arose from a duplication of an ancestral *ZmASN4* gene after the divergence of the *Panicoideae* from the *Pooideae* and even after the divergence of maize and sorghum. *ZmASN2* (Zm00001d044608) showed highest similarity to *TaASN1* and *TaASN2*, with 77.7–78.1% nucleotide sequence identity with *TaASN1* and 75.9–76.0% with *TaASN2*. The lack of a second gene (i.e., a *TaASN2* equivalent) confirms that the gene duplication event that led to the separation of the ancestors of *TaASN1* and *TaASN2* happened after the divergence of the Panicoideae and Pooideae, during the evolution of the Triticeae. As with sorghum, the maize group 1 gene has diverged less from the group 4 genes than is the case in brachypodium and the Triticeae, and the phylogenetic analysis placed it alongside *SbASN1* in the group 4 cluster (Figure 2). *ZmASN1* (Zm00001d045675) was most similar to the *TaASN3s* (83.0–84.0% nucleotide sequence identity), the presence of a single gene again confirming that the duplication event that gave rise to the *ASN3.1* and *ASN3.2* genes occurred later, during the evolution of the Triticeae.

The *SbASN* gene on chromosome 5, designated *SbASN1*, showed 87.3% similarity to the *ZmASN2* gene on chromosome 3 (Table S1, Matrix 2.7). *SbASN3* (chromosome 10) showed 95.2% to *ZmASN1* (chromosome 9), with *SbASN4* (chromosome 1) showing 94.4% to *ZmASN3* (chromosome 1) and 84.7% to *ZmASN4* (chromosome 9). Despite some differences in gene positioning, therefore, the sorghum and maize genes showed a high level of similarity.

*SbASN3* has been shown to be the most highly expressed asparagine synthetase gene overall in sorghum seeds, but *SbASN1* is the most highly expressed in the embryo (Davidson et al., 2012; Makita et al., 2015). Indeed, while *SbASN3* and *SbASN4* are expressed throughout the plant, *SbASN1* is embryo‐specific (Davidson et al., 2012), meaning that it has an even narrower tissue‐specific expression than *TaASN2* in bread wheat. The group 3 gene of maize (annotated as *ZmASN1*) is also the most highly expressed asparagine synthetase gene in the grain of maize (Chen et al., 2017; Zhan et al., 2015), but it is also highly expressed in leaves (Baute et al., 2015), seedlings (Chang et al., 2012) and 5‐day‐old caryopses (Pang et al., 2019). One of the group 4 genes (annotated as *ZmASN3*) is also highly expressed in 5‐day‐old caryopses (Pang et al., 2019). The group 1 gene (annotated as *ZmASN2*) was found to be expressed at very low levels in all of these studies, but so far data are not available on its expression later on in seed development.

## 6 RICE (*ORYZA SATIVA*)

Rice is one of 27 species in the *Oryza* genus. Within these 27 species there are 11 genome types, six of which are diploid (*n* = 12; genomes AA, BB, CC, EE, FF and GG) and five of which are tetraploid (*n* = 24; genomes BBCC, CCDD, HHJJ, HHKK and KKLL). The *Oryza* genus is part of the Ehrhartoideae (also known as Oryzoideae) subfamily, which diverged from the line that gave rise to the Panicoideae and Pooideae between 40 and 54 million years ago. Cultivated rice subsp. *indica* and *japonica* are both diploids (AA genome).

Two *ASN* genes were identified in reference genomes for both *indica* and *japonica* subspecies (Table 4) (Kawahara et al., 2013; Yu et al., 2002), one located on chromosome 3 (BGIOSGA010942 and Os03g0291500 for *indica* and *japonica*, respectively), which has previously been annotated as *OsASN1* by the Rice Annotation Project (Sakai et al., 2013), and the other on chromosome 6 (BGIOSGA021489 and Os06g0265000 for *indica* and *japonica*, respectively), previously annotated as *OsASN2* (Sakai et al., 2013). The BGIOSGA021489 (*indica*) and Os06g0265000 (*japonica*) genes have identical nucleotide sequences and encode identical 591 amino acid proteins. However, BGIOSGA010942 (*indica*) and Os03g0291500 (*japonica*) differ slightly, with BGIOSGA010942 missing 42 bp at the 5′ end of the coding sequence, resulting in BGIOSGA010942 encoding a 590 amino acid protein compared with the protein of 604 amino acids encoded by Os03g0291500. Only 65.3% nucleotide sequence identity was seen between Os03g0291500 and BGIOSGA021489/Os06g0265000, and 64.1% identity between Os06g0265000 and BGIOSGA010942/Os03g0291500 (Table S1, Similarity Matrix 2.8).

Comparison of the rice genes with the bread wheat genes showed BGIOSGA021489 and Os06g0265000 to be most similar to the *TaASN3s*, with similarities of 86% or above, while BGIOSGA010942 and Os03g0291500 showed the highest similarity to the *TaASN4s* (83.5–86.3%) (Figure 2 and Table S1 [Similarity Matrix 2.9]). In other words, rice contains no group 1 or group 2 asparagine synthetases.

Of the two genes that rice does have, the group 3 gene (annotated as *OsASN2*) has been shown to be the more highly expressed in seeds, shoots and the root tips of developing seedlings (Davidson et al., 2012; Reynoso et al., 2018; Sakai et al., 2011; Zhang et al., 2014), while the group 4 gene (annotated as *OsASN1*) shows greater expression in anthers and carpels (Zhang et al., 2014). No data are available on expression in developing seeds.

## 7 ASSIGNMENT OF ALL THE ASPARAGINE SYNTHETASE GENES TO GROUPS

**FIGURE 3:**
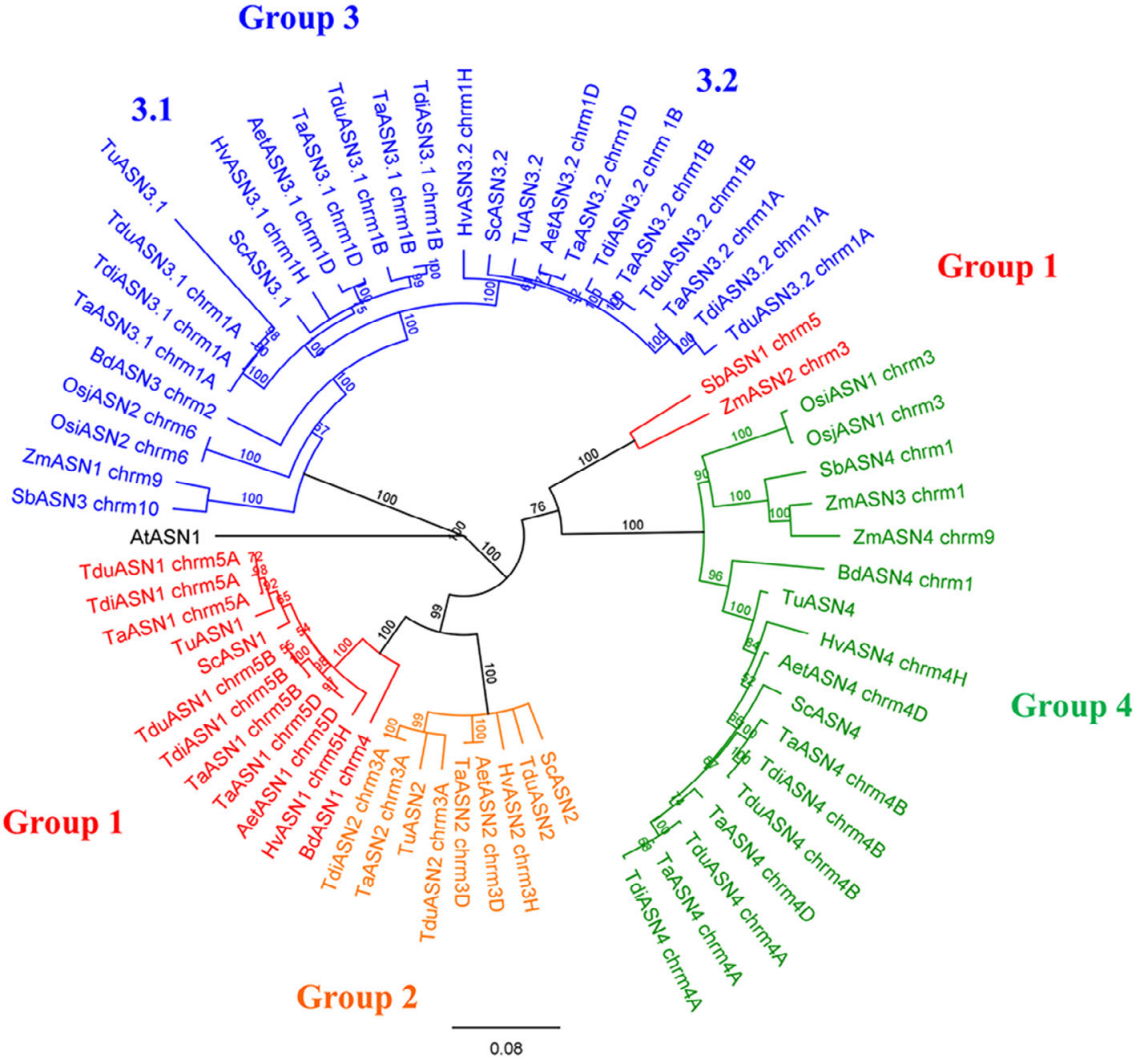
Phylogenetic tree of all the asparagine synthetase genes analysed in this study, shown with prefixes Ta (bread wheat; *Triticum aestivum*), Tdi (emmer wheat; *Triticum dicoccoides*), Tdu (pasta wheat; *Triticum durum*), Tu (einkorn wheat; *Triticum urartu*), Aet (Tausch's goatgrass; *Aegilops tauschii*), Hv (barley; *Hordeum vulgare*), Sc (rye; *Secale cereale*), Zm (maize; *Zea mays*), Sb (sorghum; *Sorghum bicolor*), Bd (brachypodium; *Brachypodium distachyon*) and Os (rice; *Oryzae sativa*). Genes are listed in Tables 2, 3, 4 and S1. The branch labels show the consensus support in the clade from bootstrapping, shown as a percentage. The scale bar represents the length of the branches, expressed in units of substitutions per site of the sequence alignment. The arabidopsis (*Arabidopsis thaliania*) gene, *AtASN1*, was used as an outgroup to anchor the tree. Note that as in Figure 2 the maize and sorghum group 1 genes actually cluster with the group 4 genes, but are closely related to both groups and are assigned to group 1 based on similarity matrices (Table S1, Similarity Matrices 2.4 and 2.6)

**FIGURE 4:**
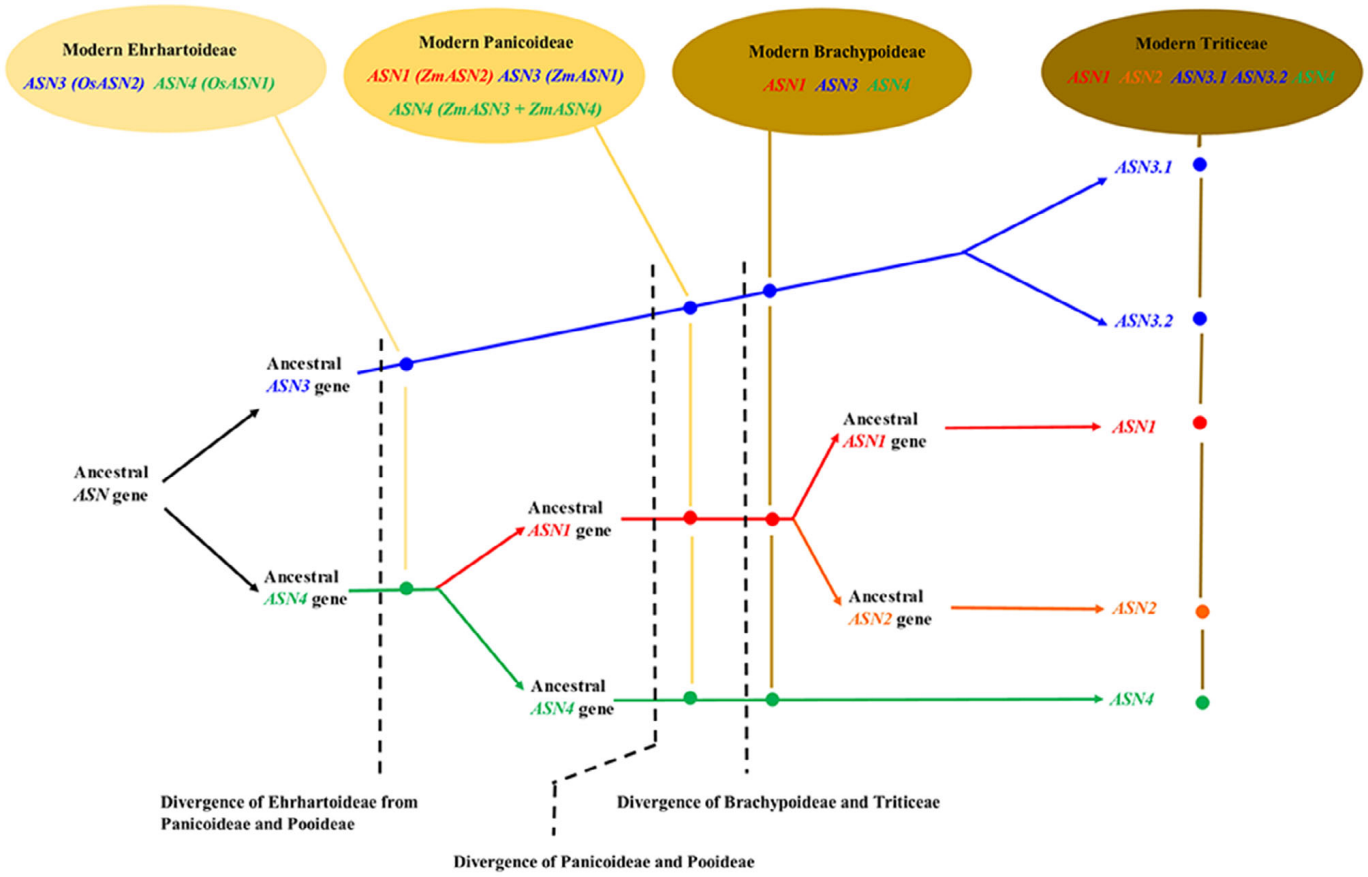
Diagram representing the evolution of asparagine synthetase genes in the Triticeae, Brachypodiae, Panicoideae and Ehrhartoideae

Confirmation of the assignment of the different genes to groups 1–4, with group 3 subdivided into 3.1 and 3.2, was achieved by compiling a phylogenetic tree of all the genes. The groups were numbered to fit the annotation of the wheat genes, with *TaASN1* in group 1 and so on. The tree is shown in Figure 3. The analysis confirmed that the family structure of five genes, in groups 1, 2, 3 (separated into 3.1 and 3.2) and 4, is unique to the Triticeae tribe, with brachypodium, maize and sorghum lacking a group 2 gene and having only one group 3 gene (although maize has two group 4 genes and several truncated genes), and rice having only group 3 and group 4 genes. Based on these observations, we propose Figure 4 as a representation of the evolution of the gene family.

## 8 CONCLUSIONS AND CONSTRUCTION OF AN EVOLUTIONARY PATHWAY FOR CEREAL ASPARAGINE SYNTHETASE GENES

The review of cereal genome data highlighted the inconsistency in the nomenclature of the asparagine synthetase gene family across the species in which the genes have already been annotated. We have annotated the five bread wheat (*T. aestivum*) genes as *TaASN1*, *TaASN2*, *TaASN3.1*, *TaASN3.2* and *TaASN4*, with corresponding annotation for the genes from the other wheat species. The nomenclature assigned previously to the five corresponding barley genes was *HvASN1*–*HvASN5* (Avila‐Ospina et al., 2015), but the barley gene family has the same structure as the wheat gene family, and the nomenclature is readily reconcilable if *HvASN4* is annotated as *HvASN3.2* and *HvASN5* is annotated as *HvASN4*.

*TaASN1* and *TaASN2* were named as such because they were the first asparagine synthetase genes to be identified in wheat (Wang, Liu, Sun, & Zhang, 2005). The same is true for the maize and rice genes, but the maize gene annotated as *ZmASN1* is a Group 3 gene and *ZmASN2* is a group 1 gene, while *ZmASN3* and *ZmASN4* are both group 4 genes, with no genes in group 2 (Figures 2 and 3). For rice, *OsASN1* is a group 4 gene while *OsASN2* is a group 3 gene, with no genes in groups 1 or 2. Genes of the other species that were identified had not been annotated previously, and we have assigned names based on the groupings.

We propose that the evolutionary development of the gene family began with an initial gene duplication that gave rise to the ancestors of the group 3 and group 4 genes (Figure 4). The Ehrhartoideae then diverged from the line that gave rise to the Panicoideae and Pooideae and the gene family did not expand further in the Ehrhartoideae. A duplication of the *ASN4* gene then occurred before the divergence of the Panicoideae and Pooideae, giving rise to ancestors of the group 4 and group 1 genes in those subfamilies. Both the Panicoideae and the Brachypoideae have three genes, *ASN1*, *ASN3* and *ASN4*, although in maize (but not sorghum) the *ASN4* gene has duplicated to give two group 4 genes. Two further gene duplications must have occurred in the ancestor of the Triticeae, firstly *ASN1* giving rise to *ASN1* and *ASN2*, then *ASN3* giving rise to *ASN3.1* and *ASN3.2*. The Triticeae therefore all have five asparagine synthetase genes per haploid genome, except that some hexaploid bread wheat (*T. aestivum*) varieties and emmer wheat (*T. dicoccoides*) have lost the B genome *ASN2*. The group 1 gene of brachypodium and the group 1 and 2 genes of the Triticeae have diverged further away from the group 4 genes than the maize or sorghum group 1 genes have. Indeed, the maize and sorghum group 1 genes are very closely related to the group 4 genes, and marginally clustered with the group 4 genes in the phylogenetic analyses (Figures 2 and 3).

We used arabidopsis gene *AtASN1* (Lam et al., 1994) to anchor the trees generated in our analyses. Arabidopsis actually has three asparagine synthetase genes (*AtASN1*, *AtASN2* and *AtASN3*) (Lam, Hsieh, & Coruzzi, 1998). Gaufichon et al. (2010) proposed that asparagine synthetase genes could be divided into two classes, I and II, with *AtASN1* in class I and *AtASN2* together with *AtASN3* in class II. Duff (2015) proposed a third class, class III, based on a phylogenetic analysis of asparagine synthetase genes from both monocotyledonous and dicotyledonous species in which a cluster of cereal genes emerged as a separate clade. That class corresponds to group 3 in our analysis, while class II roughly corresponds to our group 4. Rice is shown as having class II and III genes (groups 3 and 4 in our analysis) but no group 1. However, the barley genes grouped differently to our analysis, with three of them shown as group 1. This may be because nucleotide sequence data for many more cereal genes are now available. It is now clear that the cereal gene family has evolved considerably since the divergence of monocots and dicots and does not fit well within a broader classification that attempts to include all of the plant genes. For example, in our analyses it proved unhelpful to include more than one arabidopsis gene in the phylogenetic trees because the arabidopsis genes did not cluster with the cereal groups (not shown).

Duff (2015) proposed that the three classes of asparagine synthetases emerged before the divergence of monocots and dicots, but that many species lost some genes and saw duplications in others. If this model were correct it would mean that rice once had an *OsASN1* gene but lost it. However, this conclusion is arrived at again by trying to fit the cereal genes into a broader classification that no longer looks convincing to us. We propose that the evolution of plant asparagine synthetase genes into the gene families seen today occurred after the separation of monocots and dicots, and that the model shown in Figure 4 is a better fit for the cereal data now available.

The presence of a B genome *ASN2* gene in some hexaploid wheat (*T. aestivum*) varieties (genomes AABBDD) and in pasta wheat (*T. durum*; genomes AABB) but the absence of a B genome *ASN2* gene in emmer wheat (*T. dicoccoides*; genomes AABB) raises questions about bread and pasta wheat ancestry. If emmer wheat is the donor of the A and B genomes of bread and cultivated pasta wheat, then all hexaploid wheat varieties and pasta wheat might be expected to have inherited the B genome *ASN2* deletion. In our view, the most likely explanation is that some emmer wheat genotypes have a B genome *ASN2* gene while some lack it, and that the hybridisation event that produced bread wheat occurred more than once, involving B genome donors with and without a B genome *ASN2* gene. Another possible but in our view less likely explanation is that the B genome *TdiASN2* gene was present when the hybridisation event that produced hexaploid wheat occurred, but that emmer wheat and some hexaploid wheat genotypes subsequently lost it. A third possibility is that some bread and pasta wheat genotypes regained a B genome *ASN2* gene through an introgression from a related species, such as a wild wheat or rye.

Another important question, given the link between free asparagine concentrations, acrylamide formation during baking and processing, and food safety, is the implications that differences in the asparagine synthetase gene family have for free asparagine concentrations in the grain of the different species. In bread wheat, *TaASN2* is expressed specifically in the grain, with much higher expression in the embryo than the endosperm (Curtis et al., 2019; Gao et al., 2016), and its expression in the grain at mid‐development is far higher than the expression of any of the genes in any other tissue. Biochemical analyses have shown little difference in activity of the ASN1 and ASN2 enzymes (Xu et al., 2018), making the *TaASN2* gene almost certainly the most important in determining the amount of free asparagine that accumulates in wheat grain. The A genome *TaASN2* is much more highly expressed than the D genome gene (Curtis et al., 2019), but the relative expression of the B genome gene when it is present is not yet known.

Rice lacks homologues of both *TaASN1* and *TaASN2*, but little information is available on the concentration of free asparagine in rice grain. The fact that it only has two genes may mean that there is less scope for genetic interventions to reduce free asparagine accumulation: T‐DNA insertion mutants and CRISPR‐Cas edited lines lacking a functional version of the gene that we annotate as *OsASN4*, for example, showed effects on plant height, root length, and tiller number compared with wild type (Luo et al., 2019). Note that this gene was annotated as *OsASN1* by the authors of that study. Maize also lacks a group 2 gene, but it does contain a group 1 gene. Expression analyses of the maize gene family have shown the group 1 and group 3 genes (previously annotated as *ZmASN2* and *ZmASN1*, respectively) to be expressed in the seed, but expression in other tissues is higher (Todd et al., 2008). In other words, the high asparagine synthetase gene expression in the grain of wheat, accounted for mainly by *TaASN2*, has not been observed in maize. In addition, biochemical analyses have shown the reactions catalysed by the wheat asparagine synthetases, ASN1 and ASN2, to proceed much more rapidly than has been reported for the maize enzymes (Duff et al., 2011; Xu et al., 2018). However, a study that measured free asparagine concentrations in a range of cereal species found maize to be lower than rye but higher than barley, pasta wheat or bread wheat (Žilić et al., 2017).

Clearly, more research is required on the role of different asparagine synthetase genes in controlling the concentration of free asparagine in maize and rice grain, and more data are required generally on the concentrations of free asparagine in different cereal grains. Eighteen years after the discovery of acrylamide in food and the identification of free asparagine as its precursor, the data on free asparagine concentrations in these major food crops are surprisingly sparse. These data will be required along with much more data on and improved understanding of the tissue‐specific and developmental expression patterns of different gene family members in the various species before relationships can be drawn between gene family structure, expression patterns and relative free asparagine concentrations.

## Supporting information


**Appendix** S1: Reference numbers for asparagine synthetase genes analysed in the study. Matrices 1.1–1.15, 2.1–2.10 and 3.1.
